# Cost-benefit analysis of universal pool fencing for Texas households with young children

**DOI:** 10.1186/s40621-026-00706-0

**Published:** 2026-08-17

**Authors:** James Rhodes, Linh Nguyen, Rohit P. Shenoi

**Affiliations:** 1 Department of Pediatrics, Texas Children’s Hospital, Baylor College of Medicine, 6621 Fannin St., Suite W 1985, Houston, TX 77030 USA; 2 Department of Innovation and Quality, UT Health Houston McGovern Medical School, 6410 Fannin St, TX 77030 Houston, USA; 3 Division of Emergency Medicine, Department of Pediatrics, Texas Children’s Hospital, Baylor College of Medicine, 6621 Fannin St., Suite A 2210, Houston, TX 77030 USA

**Keywords:** Pediatric drowning, Drowning prevention, Swimming pools, Isolation Pool Fencing, Injury Prevention, Cost-benefit analysis, Public health economics, Residential pool safety

## Abstract

**Background:**

Texas bears a disproportionate burden of pediatric drowning in the United States. Functional pool fencing prevents pediatric drownings, but its economic impact on drowning-related costs remains unclear. We estimated the potential cost savings of implementing a universal pool fencing program for households with young children.

**Methods:**

We conducted a cost-benefit analysis of universal pool fencing for Texas households with one or more children ages 1–9 years. Drowning burden was estimated from Texas data with a case-fatality rate of 1:6. Direct medical and lifetime indirect costs per patient and caregiver were derived from hospital discharge data and literature. Prevented drownings were estimated using a population-wide risk model incorporating fence effectiveness and current fence coverage (percent of homes with functional fences). Average pool fence cost was obtained from industry experts, assuming a 10-year lifespan and no maintenance costs. Population and household estimates were derived from U.S. Census data to determine the number of fences required. Total program costs were calculated by multiplying the number of fences required by the average cost per fence. Annual cost savings were calculated from prevented cases and per-case costs. Benefits over a 10-year period were estimated as the present value of annual savings discounted at 5%. Net benefit was defined as 10-year benefits minus total fencing program costs.

**Results:**

The annual fatal and non-fatal drowning burden in Texas among children ages 1–9 years is 264 cases. With 76% fence effectiveness and 38% current fence coverage, an estimated 174 drownings could be prevented annually. The average cost per drowning is $337,435 (direct medical: $60,860; lifetime indirect: $276,575). With 7% of Texas households having a pool, an estimated 98,905 new fences are required at a cost of $3,343 each, totaling $330,642,758 in program costs. Annual cost savings are $58,713,690, corresponding to $453,371,551 in 10-year benefits and a net benefit of $122,728,793.

**Conclusions:**

Universal pool fencing in Texas could prevent pediatric drownings and yield significant economic savings. These findings support stronger residential pool safety standards, including statewide mandates, robust inspection and enforcement tied to permitting, and financial incentives such as rebates or insurance discounts to offset installation costs.

## Background

Drowning is the leading cause of death in children ages 1–4 years and the second leading cause of unintentional injury death in children ages 1–14 years [1]. Between 2018 and 2023, nearly 4,025 children ages 1–14 years died from unintentional drowning, with 3,450 (86%) of these deaths among children ages 1–9 years [1]. This impact is particularly evident in Texas, which had the highest total number of fatal pediatric drownings among children ages 0–19 years from 2018 to 2022 [1]. 

Toddlers and young children primarily drown in swimming pools, where inadequate or improperly functioning barriers are a key factor [2, 3]. Pediatric pool drownings also impose a substantial economic burden, including both direct medical costs (emergency care, intensive care, and rehabilitation) and indirect costs (productivity loss and long-term caregiving), along with profound emotional and quality-of-life impact for patients and caregivers. In these settings, functional pool fencing is an effective drowning prevention strategy. Four-sided isolation fencing (separating the pool from the home and yard with a self-closing and self-latching gate) significantly reduces drowning risk compared with three-sided perimeter fencing that permits direct access from the home [4]. Despite recommendations from the American Academy of Pediatrics (AAP) and the U.S. Consumer Product Safety Commission (CPSC), four-sided isolation fencing remains underutilized, with nearly two-thirds of parents who own pools or spas lacking it [5–7]. Yet many states, including Texas, still allow three-sided fencing despite clear evidence it is less effective [8]. 

Although functional pool fencing effectively reduces drowning risk, its potential economic benefit in preventing pediatric drownings is not well quantified. This study assessed the cost-benefit of universal functional pool fencing in Texas.

## Methods

We conducted a cost-benefit analysis of implementing a hypothetical universal swimming pool fencing program for Texas households with young children. Functional pool fencing is defined as a code-compliant, properly installed, four-sided isolation barrier at least four feet high with a self-closing and self-latching gate that prevents young children from gaining unintended access to the pool. The analysis was performed from a societal perspective, comparing the upfront total program cost (fence materials and installation) with the economic benefits of preventing pediatric drowning events among children ages 1–9 years, using data and parameters from several state databases. We report our analysis according to the consolidated health economic evaluation reporting standards (CHEERS) checklist [9]. 

### Target population and prevented drownings

Children ages 1–9 years were selected in this study because drowning risk in residential swimming pools is highest in this age group. Texas households with one or more children ages 1–9 years were identified using the 2024 American Community Survey Public Use Microdata Sample accessed through IPUMS USA [10]. Person-level records were restricted to Texas residents living in housing units (STATEFIP = 48; GQ ≤ 2), and children ages 1–9 years were identified using the age variable (AGE). Records were grouped by household serial identifier (SERIAL) to ensure each household was counted once. Population estimates were generated by summing household weights (HHWT) across qualifying households, yielding an estimated 2,278,934 Texas households with at least one child aged 1–9 years.

The number of households with residential swimming pools was estimated by applying a pool ownership rate to households with children ages 1–9 years. Prior estimates indicate that approximately 7% of Texas single-family households have residential swimming pools [11]. This proportion was applied to households with children ages 1–9 years to estimate the number of single-family households with both a swimming pool and a child in this age group. Based on drowning data from Harris County, Texas, current fence coverage rate was assumed to be 38%, defined as the percentage of households with functional pool fences [12]. The number of new fences required was estimated by multiplying the number of pools in households with children ages 1–9 years by (1 – coverage rate).

The mean annual number of swimming pool drowning fatalities among children ages 1–9 years in Texas from 2018 to 2023 was obtained from the Centers for Disease Control and Prevention (CDC) WONDER database [13]. The estimated annual burden of fatal and non-fatal drowning events was calculated using a pediatric drowning case-fatality rate of 1:6 [14]. Using a relative risk (RR) estimate of 4.1 from the literature, effectiveness of four-sided isolation pool fencing in preventing drowning was assumed to be 76% [15, 16]. Prevented drownings were estimated using a population-wide risk re-weighting model equivalent to the potential impact fraction described by Morgenstern, Chan, and Mitsakakis, in which expected post‑intervention events equal the baseline burden multiplied by the ratio of counterfactual to observed average risk [17–19].

$$\begin{gathered}Prevented\;Drownings=D_{pre}-D_{post}\\=D_{pre}[1-\frac{\frac{1}{RR}}{(1-Coverage)+\frac{Coverage}{RR}}]\end{gathered}$$

where D_pre_ is the observed number of annual drownings among children ages 1–9 years in Texas pre-intervention, D_post_ is the estimated number post-intervention, coverage is the proportion of households with functional fencing, and RR is the relative risk of unfenced versus fenced pools, with unfenced pools treated as the reference category (RR = 1).

### Program costs

Average fence size and cost were obtained from six Texas pool companies. Based on expert recommendations, a medium-sized pool was assumed to require 110 linear feet of mesh fencing. A medium-sized pool was selected because local pool companies identified this size as the most common residential pool model. The average cost per pool fence included materials, a self-closing and self-latching gate, labor, and an 8.25% sales tax. Mesh fencing was selected for its safety and regulatory compliance, ease of installation, cost-effectiveness, removability, and low maintenance due to its durability [20]. A 10-year fence lifespan with no maintenance costs was assumed, reflecting the period during which young children require close supervision and remaining well within the typical lifespan of mesh pool fencing (15–20 years) [21–23]. Total program costs were calculated by multiplying the number of new fences needed by the average cost per fence.

### Economic benefits

Economic benefits were defined as avoided drowning-related costs per prevented drowning event, including direct medical costs (inpatient and outpatient) and lifetime indirect costs from patient and caregiver productivity loss. Lifetime indirect costs were discounted to the time of the drowning event to reflect the present value of future productivity losses per case. Costs of unintentional drownings in swimming pools were estimated among children ages 1–9 years treated in Texas hospitals from 2016 to 2022. Medical cost data were obtained from the Texas Health Care Information Collection (THCIC) for 2016–2022 and included hospital inpatient (IP) and hospital-based outpatient (OP) services, length of stay, patient demographics (age group, sex, race, ethnicity, and insurance), discharge diagnoses, and discharge status [24]. Hospital encounters for unintentional fatal and non-fatal drownings were identified using the International Classification of Diseases, Tenth Revision, Clinical Modification (ICD-10-CM) diagnosis codes indicating drowning in a swimming pool: W65, W67, W68, W16.0, and W22.041. Study of the burden of unintentional drownings was approved by the Institutional Review Boards of Baylor College of Medicine and the University of Texas Health Science Center at Houston.

Direct medical costs (inpatient and outpatient costs) and lifetime indirect costs (patient and caregiver productivity loss) were calculated for patients hospitalized after an unintentional drowning in a swimming pool [25]. Medical costs included hospital stays, outpatient visits, physician services, diagnostic tests, drugs and medical supplies, and ambulance transport. Lifetime indirect costs reflected productivity loss among patients with drowning-related disability and caregivers providing long-term care. Indirect costs were estimated using the human capital approach, assuming a 20% long-term disability rate [26–29]. Productivity loss from death and the value of statistical life were not included because assigning monetary value to a life lost is controversial and complex [30]. All costs were discounted at 5% and reported in 2022 US dollars [31, 32]. 

### Net benefits and return on investment

The analysis used a 10-year time horizon aligned with the expected lifespan of the pool fence and the period during which young children require close supervision. Fence installation costs were assumed to occur at the time of program implementation, whereas benefits accrued annually over the 10-year period. Annual cost savings from prevented drowning events were estimated by multiplying the annual number of prevented drownings by average cost per drowning case, then discounting to the time of program implementation at a 5% annual rate. Total benefits were calculated as the present value of discounted annual benefits over the intervention lifespan. Net benefit was defined as 10-year benefits minus total program costs and return of investment (ROI) as net benefit divided by total program costs.

Sensitivity analyses (SA) were conducted to assess the robustness of results to uncertainty in key parameters, including fence effectiveness (RR, 2.0–4.1), baseline fence coverage (38%;76%), and fence installation costs ($2,619;$4,068) [15, 16, 33]. The SA also included scenario analyses in which extreme medical costs were replaced with lower percentile values: scenario 1, the top 1% replaced by the 99th percentile value; scenario 2, the top 5% replaced by the 95th percentile value; and scenario 3, the top 10% replaced by 90th percentile value. An additional SA scenario estimated indirect costs for patients and caregivers at $269,392 when long-term disability was identified by ICD-10-CM codes for hypoxic-ischemic injury in our dataset, compared with $276,575 when applying a 20% disability rate.

## Results

From 2018 to 2023, there were 264 fatal swimming pool drownings (44 fatal drownings per year) in Texas among children ages 1–9 years. Using a pediatric drowning case-fatality rate of 1:6, the estimated annual burden of fatal and non-fatal drowning events was 264 cases. (Table 1)

**Table 1:** Model parameters and data sources used in cost-benefit analysis

Parameters	Value	Source
Annual drowning burden (fatal + nonfatal) Annual fatal swimming pool drownings Total number of drowning events per fatality	264 44 6* (*Using a 1:6 case fatality rate)	Calculated CDC Wonder (2018–2023) *Shenoi et al.*,* Pediatr Emerg Care (2025)*
Isolation pool fence effectiveness rate	76%* (*Using a RR estimate of 4.1)	*Pitt et al.*,* Med J Aust (1991)*
Current coverage rate	38%	*Shenoi et al.*,* Inj Epi (2025)*
Total costs per pediatric patient Mean inpatient costs (1–9 years) Mean outpatient costs (1–9 years) Mean Indirect costs (1–9) to patient + caregiver	$337,435 $57,543 $3317 $276,575	Calculated THCIC (2016–2022) THCIC (2016–2022) *Nguyen et al.*,* Inj Prev (2025)*
Average cost for a mesh pool fence	$3343	Local pool companies
Number of Texas households with one or more children ages 1–9 years	2,278,934	IPUMS USA (2024)
Percent of Texas households with residential swimming pool	7%	*Shenoi et al.*,* Inj Prev (2015)*
Number of New Fences Required	98,905	Calculated
Annual number of prevented drownings	174	Calculated
Annual cost savings	$58,713,690	Calculated
Total fence installation costs	$330,639,415	Calculated
10-year time horizon cost savings	$453,371,551	Calculated
Net Benefit	$122,732,136	Calculated
Return on Investment	37%	Calculated

The average cost per drowning case in Texas is $337,435 (medical costs: $60,860; lifetime indirect costs: $276,575). Using IPUMS USA household counts, a 7% pool ownership rate, and a 38% functional fencing coverage rate, 98,905 new fences are required for a universal pool fencing program, assuming one fence per pool. With a 76% fence effectiveness rate, functional pool fencing would prevent an estimated 174 annual drownings among children ages 1–9 years in Texas. (Table 1)

With an average mesh fence cost of $3,343 ($2,619–$4,068) for a medium-sized pool, installing functional fencing for the remaining 62% of homes would total $330,642,758 in program costs. Thus, preventing 174 drowning cases would yield annual cost savings of $58,713,690, corresponding to $453,371,551 in 10-year benefits. The projected net benefit over 10 years is $122,728,793, with a ROI of 37%. (Table 1)

**Fig. 1:**
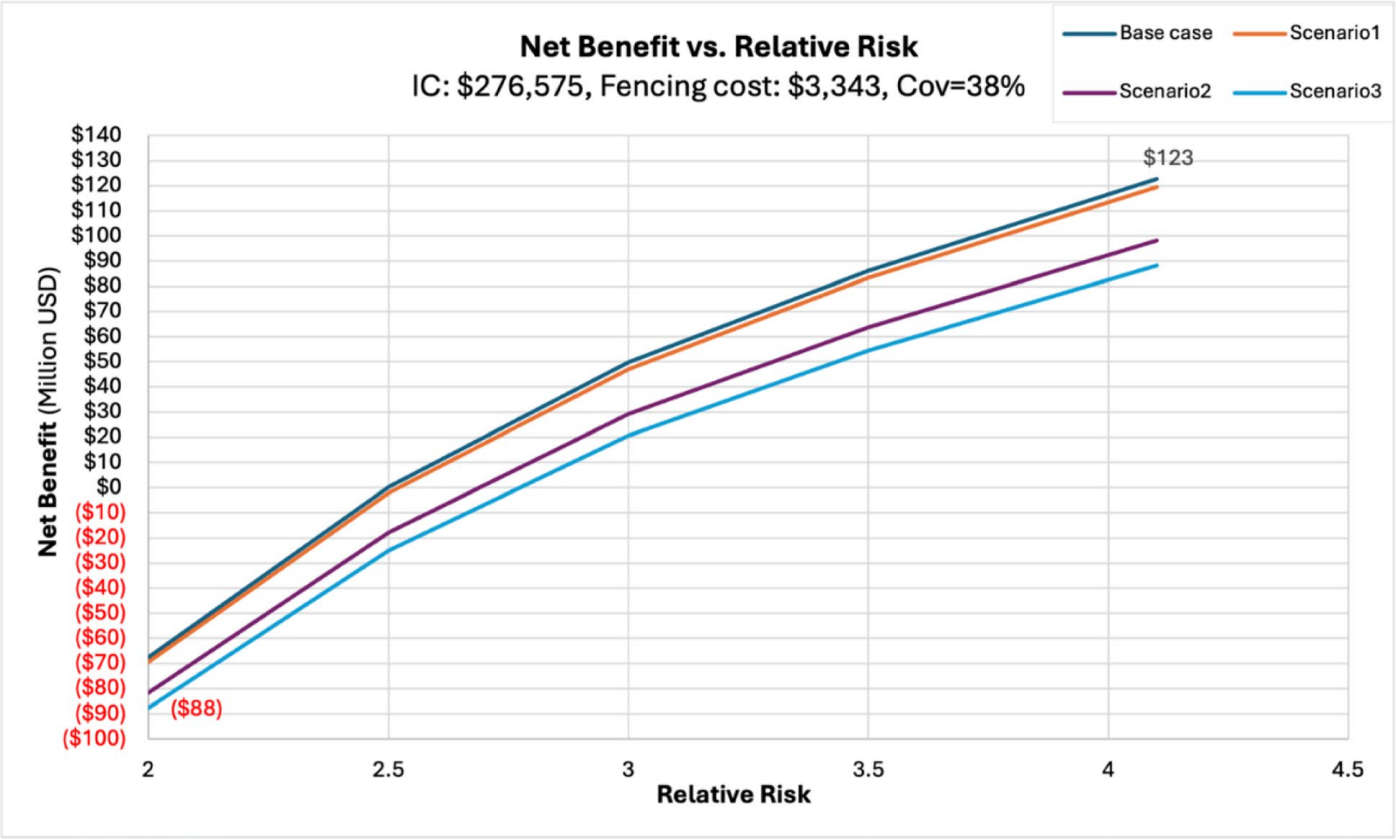
Net economic benefit of universal pool fencing across relative risk scenarios. Net benefit of universal pool fencing across RR assumptions. Sensitivity analyses by RR (2.0–4.1) and medical cost inputs, using a base-case fencing coverage of 38% and mean fencing and indirect costs. Scenario analyses replace the highest-cost cases with the 99th (Scenario 1), 95th (Scenario 2), and 90th (Scenario 3) percentile values

**Fig. 2:**
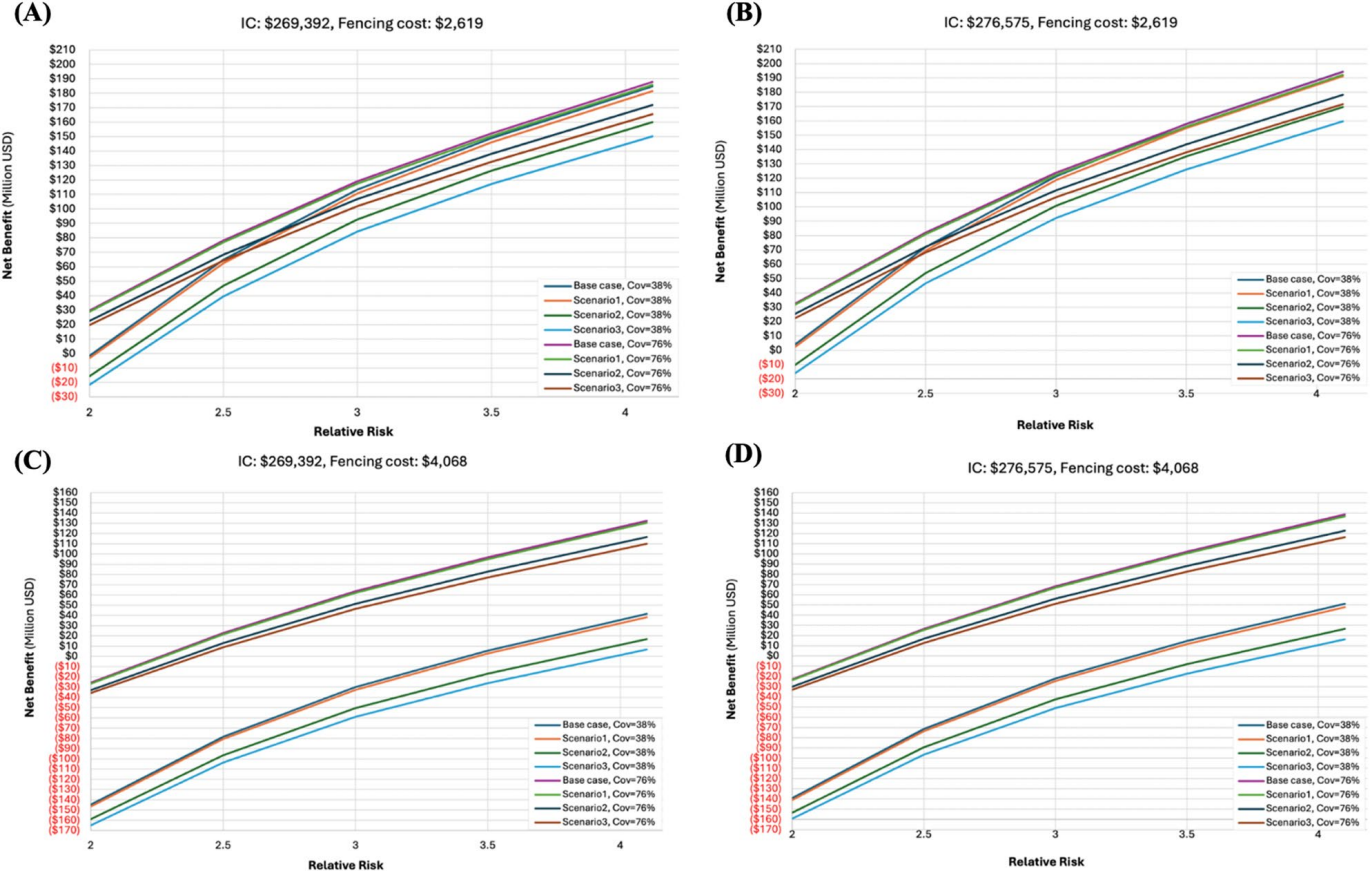
Sensitivity analysis of net economic benefit across relative risk, installation costs, and coverage scenarios. Net benefit of universal pool fencing across RR assumptions. Sensitivity analyses vary by RR (2-4.1), coverage rate (38%;76%), fencing costs ($2,619; $4,068), including an alternative estimate of indirect costs using ICD-10–identified hypoxic–ischemic injury ($269,392) compared with a 20% disability assumption ($276,575). Scenario analyses replace the highest-cost cases with the 99th (Scenario 1), 95th (Scenario 2), and 90th (Scenario 3) percentile values

Across extensive sensitivity analysis, net benefit of universal pool fencing remained positive under most plausible parameter combinations, with results primarily driven by assumptions about fence effectiveness (RR), coverage, and installation costs. (Table 2) Net benefits increased monotonically with higher assumed risk reductions associated with four-sided fencing (RR, 2.0–4.1). With a fence coverage rate of 38%, net benefits were positive at moderate to high RR values (Fig. 1). With a higher fence coverage rate (76%), net benefits were positive across nearly all scenarios, driven by lower program costs. As expected, higher fencing costs attenuated net benefits but did not eliminate positive benefits at higher RR assumptions. (Fig. 2) Results were similar under both assumptions for lifetime indirect costs. (Figures 1 and 2)

**Table 2:** Model parameters and data sources used in sensitivity analysis

Parameters	Ranges	Source
Relative Risk	2.0 4.1	*Logan et al. Pediatrics (1998)* *Pitt et al.*,* Med J Aust (1991)*
Cost of fencing	$2619 $4068	Local pool companies
Coverage Rate	38% 76%	*Shenoi et al.*,* Inj Epi (2025)* *Gilchrist et al.*,* IJARE (2008)*
Mean Inpatient costs (1–9 years)		
Base Case Scenario 1 Scenario 2 Scenario 3	$57,543 $55,262 $39,640 $32,355	THCIC (2016–2022)
Mean Outpatient costs (1–9 years)		
Base Case Scenario 1 Scenario 2 Scenario 3	$3317 $3267 $2920 $2767	THCIC (2016–2022)
Mean Indirect costs (1–9) to patient + caregiver		
Presence of hypoxic-ischemic injury Applying a 20% disability rate	$269,392 $276,575	ICD-10 codes *Bell et al. (1985); Bratton et al. (1994); Peden et al. (2008)*

## Discussion

Our cost-benefit analysis demonstrates that implementing universal isolation pool fencing in Texas households with young children aged 1–9 years could prevent 174 pediatric drownings annually and yield over $58 million in annual cost savings for the state. Over a 10-year period, these savings correspond to more than $453 million in discounted benefits. These findings reinforce the public health importance of pool fencing as a drowning countermeasure and underscore the substantial economic return, roughly $1.37 saved for every $1 invested on fence installation. Importantly, these estimates do not include the potential economic benefit for the pool-fencing industry from widespread adoption, nor do they account for families with young children who visit a Texas household with children ages 1–9 years and a swimming pool, suggesting the true economic and lifesaving impact may be even greater. Additionally, this analysis assumes a conservative 10-year fence lifespan, whereas many pool safety experts estimate mesh fencing lasts 15–20 years, suggesting benefits may extend well beyond the time horizon modeled.

Our findings were robust across a wide range of plausible assumptions regarding fence effectiveness, coverage, installation costs, and indirect cost estimation. Importantly, net benefits remained positive in most scenarios, particularly with moderate to high fence effectiveness and expanded coverage, supporting the economic viability of isolation pool fencing as a population-level injury prevention strategy. The strong influence of RR assumptions reflects that fencing shifts population exposure from a higher-risk to a lower-risk state. As a result, modest changes in estimated effectiveness translate into substantial differences in the number of prevented drowning events and associated cost savings.

The medical cost estimates in our study may be higher than those reported in other studies, which employed different analytical methods. Peterson et al. edited extreme dollar values and used the difference in costs between patients who drowned and did not drown to calculate costs associated with non-fatal drowning. Other authors have used log-adjusted mean costs of care. Our study used all available values for fatal and non-fatal drowning and performed a comprehensive sensitivity analysis to compare our results with other studies. Additionally, we did not incorporate a value of statistical life (VSL) in this analysis compared with other cost savings studies [34]. Although VSL can quantify the broader societal burden of mortality and is commonly used in comprehensive cost‑benefit evaluations, our study was intentionally limited to estimating direct medical and indirect injury‑related costs. This narrower scope aligns with our objective of characterizing the economic implications of preventable drowning injuries rather than valuing mortality risk reductions. Future research focused on population‑level drowning prevention policies may appropriately incorporate VSL to capture the full societal benefits of reducing fatal drowning risks.

A key challenge in conducting a cost-benefit analysis of universal pool fencing is that there is the limited availability of data on adequate pool fencing coverage and functional fence effectiveness. Earlier telephone surveys suggested that 76% of households with pools reported adequate pool fencing [16, 35]. However, these estimates are more than two decades old. A national Safe Kids survey in 2004 found that only 39% of pool-owning families had isolation fencing, and a subsequent survey in 2016 estimated coverage as low as 18–22% based on parent report [3, 6]. Recent data from the Houston metropolitan region also indicate low coverage rates of adequate pool fencing, with estimates from 26.5% to 66% based on drowning fatality and swimming pool inspection data, respectively [33]. Our primary analysis used an estimated fencing coverage rate of 38% based on fatal drownings among children ages 1–9 years; sensitivity analyses applied the estimated fencing coverage rates of 38% and 76%. Furthermore, the best available estimates for four-sided isolation fence effectiveness are decades old, with the most recent US-specific study published in 1998 [4, 15, 16]. Our primary analysis used a relative risk estimate of 4.1 associated with isolation fencing; sensitivity analyses applied relative risk estimates ranging from 2.0 to 4.1.

Universal isolation pool fencing remains underutilized as a drowning countermeasure, in part because fencing regulations vary across Texas jurisdictions. There are municipalities in Texas, including the City of Houston, that require swimming pools and pool enclosures to be code-compliant at the time of construction; however, routine safety inspections of private single-family residential pools seldom occur and are rarely enforced [8, 36, 37]. Existing regulations that permit three-sided perimeter pool fencing allow direct access from the home and are significantly less effective than four-sided isolation fencing in preventing pediatric drowning [4]. 

Texas has demonstrated meaningful returns from prior passive injury prevention investments. A community-based smoke alarm installation initiative in Dallas yielded a net benefit of approximately $380,000 annually, translating to $128,000 saved per fire-related injury prevented and a ROI of $3.21 per $1 spent [38]. Another study in Texas estimated that child passenger safety initiatives could save $831 for each car seat misuse prevented [39]. Although differences in methodology, target populations, and time frames limit direct comparisons to our analysis, these benchmarks help contextualize the potential economic value of injury prevention strategies. Texas has a strong track record of injury prevention through programs that integrate public education, free safety equipment, and regulatory oversight across passenger safety, home safety, firearm safety, and drowning prevention [40–45]. These policy-driven injury prevention efforts reduce cost barriers, foster public-private collaboration, strengthen enforcement standards, and normalize safety behaviors. Applying similar principles to pool fencing could increase adoption of four-sided isolation fencing and reduce pediatric drowning statewide.

Cost-neutral approaches and sound fiscal stewardship are important considerations when proposing policies to expand residential pool fencing. Financial incentives, such as rebates or insurance discounts, represent a practical approach to offset fence installation costs for both homeowners and pool management companies. Rebates have been employed across multiple sectors in Texas to incentivize individual behavior, support public benefit, and stimulate economic growth. Environmental initiatives like the Texas Emissions Reduction Plan (TERP) and the Light-Duty Electric Vehicle Rebate Program illustrate how strategic public health investment can reduce environmental exposures and improve population health by promoting cleaner engines and electric vehicle adoption [46, 47]. Additional programs, including the Texas Moving Image Industry Program (TMIIIP) and the Texas Enterprise Fund (TEF), further demonstrate how state-led initiatives can drive job creation and produce measurable economic benefits [48, 49]. Collectively, these examples establish a strong foundation for advancing a state-funded residential pool fencing rebate that improves safety and economic outcomes in Texas.

Insurance-based discounts are another well-established strategy in Texas to promote home safety. Many major insurers offer voluntary discounts for common injury prevention features, such as burglar alarms, fire alarms, and smoke detectors, thereby lowering premiums for policyholders while encouraging proactive risk reduction [50]. Beyond voluntary measures, Texas law mandates insurance discounts for certain safety upgrades, including hail-resistant roofing and windstorm mitigation features (such as impact-resistant windows and storm shutters) [51–54]. In contrast, pool fencing is generally treated as a baseline underwriting requirement rather than an incentivized upgrade. Most Texas home insurance policies require pools to be enclosed by a secure fence at least four feet in height, and noncompliance can result in denied coverage or policy cancellation due to the high liability risk associated with residential pools [55, 56]. However, insurance requirements rarely differentiate between three-sided fencing and the more effective four-sided isolation fencing. This gap highlights an opportunity to align insurance practices with evidence-based injury prevention strategies. A partnership between the Texas Department of Insurance and private carriers could establish formal premium credits or state-supported incentives for four-sided isolation pool fencing, reframing pool fencing from a minimum compliance requirement to a rewarded safety measure comparable to smoke alarms or windstorm mitigation features. Overall, a coordinated approach that combines legislative enforcement with targeted financial incentives represents a sustainable framework for closing persistent gaps in drowning prevention.

This study has several limitations. First, this study incorporates drowning-related costs, drowning burden, and fencing costs but does not account for expenditures related to search and rescue efforts. Ongoing inspection and enforcement costs were also excluded because inspections of private single-family residential pools generally occur during permitting and would not represent an additional cost for newly installed fences [37]. Furthermore, this study was conducted in Texas; thus, the results may not be generalizable to other regions with different populations and waterscapes. Second, our study did not examine sociodemographic disparities in drowning-related costs, drowning burden, and swimming pool exposure. It is well established that persons belonging to racial and ethnic minorities face disproportionate barriers to accessing swimming facilities and water safety education and experience intergenerational disparities in swimming ability compared with White populations [57–59]. Additionally, families with higher household income and people of White race are more likely to own pools at single-family homes [16, 60]. Future research should examine how these factors influence drowning risk and economic burden across diverse populations. Any universal pool fencing program in Texas should prioritize children and families at greatest risk. Third, estimating lifetime indirect costs is challenging due to limited information on long-term disability following nonfatal drowning events. Variability in methods used to estimate disability prevalence may contribute to differences in cost estimates across studies. Fourth, our analysis relied on hospital discharge data, which may differ from estimates derived from insurance claims data. However, we attempted to address this limitation through sensitivity analyses. Fifth, reliable data on functional pool fencing are limited because no centralized system tracks the presence or condition of isolation fencing at residential properties. To address this limitation, we used available pool inspection records and medical examiner data as proxies [33]. Finally, while fencing is an effective drowning prevention measure, it does not eliminate risk entirely. The present analysis did not assess the role of active supervision or swimming skills training. Nevertheless, ongoing supervision, water safety education, and proper maintenance of physical barriers remain essential components of comprehensive drowning efforts.

This study has important public health policy implications. Few cost-effectiveness analyses have examined drowning prevention interventions among pediatric populations. In Bangladesh, cost-effectiveness analyses demonstrated that community-based crèches limiting unanticipated water access and swimming lessons yield substantial economic benefits, reducing drowning-related costs by approximately $812 and $85 per disability-adjusted life year (DALY), respectively [61]. Our findings underscore the potential for similar evidence-based legislative efforts to expand universal pool fencing policies at the state and national level.

Additionally, accurate and recent estimates of adequate pool fencing coverage and functional fence effectiveness in the US remain limited. Despite some limitations, the use of pool inspection, medical examiner, and child fatality review data offers a promising approach to improving population-level surveillance of fencing practices nationwide. Strengthening these data sources is critical to informing policy and evaluating intervention impact.

## Conclusion

Implementing a universal swimming pool fencing program in Texas could substantially reduce the drowning burden of young children and yield meaningful economic savings for the state. These findings support evidence-based, uniform fencing regulation and encourage the widespread adoption of four-sided isolation fencing across Texas.

## Data Availability

All datasets used in this study are publicly available.

## Declarations

### About this supplement

This article has been published as part of Injury Epidemiology Volume 13 Supplement 1, 2026: Proceedings of the 30th Annual Injury Free Coalition for Kids^®^ Conference: Forging New Frontiers: injury Prevention in Evolving Environments. The full contents of the supplement are available online at https://link.springer.com/journal/40621/volumes-and-issues/13-1/supplement.

## Abbreviations

AAP: American Academy of Pediatrics
CDC: Centers for Disease Control and Prevention
DALY: Disability-Adjusted Life Year
IP: Inpatient
ICD-10-CM: International Classification of Diseases, Tenth Revision, Clinical Modification
OP: Outpatient
RR: Relative Risk
ROI: Return On Investment
SA: Sensitivity Analyses
CPSC: U.S. Consumer Product Safety Commission
VSL: Value of Statistical Life
